# Microbiological Hazards in the Food Chain of Fish and Products, a Focus on *Klebsiella* spp.

**DOI:** 10.3390/vetsci12020133

**Published:** 2025-02-06

**Authors:** Alejandro De Jesús Cortés-Sánchez, Mayra Diaz-Ramírez, Adolfo Armando Rayas-Amor, Luis Daniel Espinosa-Chaurand, Erika Torres-Ochoa, Ma. De la Paz Salgado-Cruz

**Affiliations:** 1 Secretaría de Ciencia, Humanidades, Tecnología e Innovación (SECIHTI), Av. Insurgentes Sur 1582, Col. Crédito Constructor, Alcaldía Benito Juárez 03940, Estado de México, Mexico; 2 Departamento de Ciencias de la Alimentación, División de Ciencias Biológicas y de la Salud, Universidad Autónoma Metropolitana, Unidad Lerma, Av. de las Garzas No. 10, Col. El Panteón, Lerma de Villada 52005, Estado de México, Mexico; m.diaz@correo.ler.uam.mx (M.D.-R.); a.rayas@correo.ler.uam.mx (A.A.R.-A.); 3 Unidad Nayarit del Centro de Investigaciones Biológicas del Noroeste, Calle Dos No. 23. Av. Emilio M. González Cd. Industrial, Tepic 63173, Nayarit, Mexico; lespinosa@cibnor.mx; 4 Departamento Académico de Ingeniería en Pesquerías, Universidad Autónoma de Baja California Sur, Carretera al sur Km 5.5. Colonia el Mezquitito, La Paz 23080, Baja California Sur, Mexico; etorres@uabcs.mx; 5 Departamento de Ingeniería Bioquímica, Escuela Nacional de Ciencias Biológicas, Instituto Politécnico Nacional, Av. Wilfrido Massieu 399, Nueva Industrial Vallejo, Gustavo A. Madero 07700, Estado de México, Mexico; msalgadoc@ipn.mx

**Keywords:** food safety, food pathogens, biological hazards, quality, enterobacteria, coliforms, spoilage, biological contamination, foodborne diseases

## Abstract

Feeding is an elementary human need from which we obtain the energy and nutrients necessary for development and survival. Health heavily depends on food, which can be a means of different microbial hazards when contaminated at any stage of the food chain, compromising food safety and consumer health. Fish are considered widely produced foods (fishing or aquaculture) and are marketed worldwide; they are also a basic element of the human diet because they are a source of proteins and lipids. On the other hand, owing to their chemical properties (neutral pH and water activity), fish are highly susceptible to contamination by saprophytic and pathogenic microorganisms related to spoilage and risk to human health. Among the contaminating microorganisms in fish are bacteria of the genus *Klebsiella*, which are considered important in human and animal health worldwide due to their opportunistic pathogenicity, resistance to various antimicrobials, and association with numerous infections at the community and hospital levels, where foods such as fish and other products can serve as important sources of transmission. Therefore, this document presents a bibliographic review focused on describing, in a general way, the genus *Klebsiella* and its relationship with human health, aquatic animal health, and the safety of fish and products, as well as laboratory analysis procedures and identification of control and prevention measures of this biological hazard in fish and products to safeguard public health.

## Simple Summary

Fish is a food produced and marketed worldwide. It is considered an essential component of the human diet due to its nutritional properties. On the other hand, fish is also a food highly susceptible to contamination by various microorganisms throughout the food chain, leading to its spoilage (reduction of its shelf life) and risk to human health due to its consumption. Among the microorganisms that contaminate fish are bacteria of the *Klebsiella* genus. These bacteria are of important health interest due to the infections they cause at community and hospital levels, in addition to presenting resistance to various antimicrobials, making their treatment difficult, thus causing a negative impact on public health. This document focuses on presenting a bibliographic review focused on generally describing the *Klebsiella* genus, its relationship with human health, the health of aquatic animals, and the safety of fish and products, also indicating the procedures for its identification in the laboratory, the control and prevention measures for foodborne diseases caused by this pathogen in fish and products in order to safeguard public health.

## 1. Introduction

The quality of life and health of people are partly functions of the nutritional, hygienic, and sanitary qualities of the food they consume, which can be affected at some stage of their production chain, from the field to the consumer’s table [1]. Fish is considered an essential food in the human diet because of the contributions of various nutrients, such as unsaturated lipids, vitamins, and minerals, and the high biological value and digestibility of proteins, supplying approximately 16% of the protein consumed worldwide [2,3].

Owing to the nutritional benefits of fish, they are also highly susceptible to contamination throughout the food chain by various saprophytic and pathogenic microorganisms that lead to spoilage and a significant risk to consumer health. Thus, fish and their products are important sources of transmission of various biological agents, mainly bacteria (*Clostridium* spp., *Salmonella* spp., *Shigella* spp., *Vibrio* spp., *E. coli*, *S. aureus*, *Listeria* spp., *Klebsiella* spp., among others), causing numerous infections and poisonings derived from consumption around the world [4,5,6], particularly if the conditions in which they are produced, processed, and handled are not hygienically adequate [3,5].

*Klebsiella* spp., is a genus of bacteria of ubiquitous nature found in soil and water that is a part of the microbiota of animals, including fish intended for human consumption. These microorganisms have been identified as opportunistic pathogens associated with various hospital and community infections, becoming important clinical threats and public health challenges around the world, not only because of their incidence and negative repercussions on human and animal health but also because they present resistance to various antimicrobial agents [7]. In this context, this document presents a bibliographic review focused on the *Klebsiella* genus, its impact on human health, and aquatic animal health and its relationship with the safety of fish and products. We also discuss laboratory analysis procedures and identify control and prevention measures for these biological hazards in fish and products to safeguard public health.

## 2. Foodborne Diseases

Foodborne diseases are those diseases that occur through the consumption of foods that may contain biological or chemical contaminants of an endogenous nature or through the incorporation, at some stage, of the food chain in portions that affect the health of the consumer [1,8]. These diseases are considered major public health problems worldwide because of their morbidity, mortality, and negative impact on food purchases and sales and hospital expenses for the affected population [9,10,11]. The World Health Organization (WHO) has estimated that every year 600 million people in the world become ill from contaminated food, and 420,000 people die [12]. Approximately 250 causal agents of diseases that are transmitted through food have been identified, including bacteria, viruses, fungi, parasites, prions, toxins, chemicals, and heavy metals, and the incidence of these diseases has been increasing due to factors such as globalization in the production and marketing of food, changes in the eating habits of the population, climate change, antimicrobial resistance, population growth, the emergence of vulnerable population groups, technological advances in production, and the increased use of additives [9,10,11,13,14,15].

The most common symptoms of these diseases are vomiting, diarrhea, abdominal pain, headache, fever, neurological symptoms, and double vision, among others. They can cause complications such as chronic kidney disease, arthritis, meningitis, abortion, sepsis, Reiter’s syndrome, Guillain–Barré syndrome, or death [1,8,16]. The groups most vulnerable to these diseases are children, elderly individuals, pregnant women, immunocompromised people, and those with few economic resources, malnutrition problems, and insufficient or insufficient basic sanitation resources such as drinking water [1,16]. They also tend to predominate in areas where poor hygiene–sanitary habits and overcrowded conditions are practiced [13]. Foodborne diseases can manifest either as an infection, which occurs with the ingestion of food containing harmful live microorganisms that cause invasion, multiplication, and alterations of the host tissues, or through poisoning. This occurs when food is ingested in which the toxin found either forms in the tissues of the animal and/or plant or as a metabolite derived from the growth of microbes in the food or a chemical substance is added accidentally or intentionally at any stage of the food chain [1,10]. The biological agents frequently associated with foodborne diseases are norovirus, hepatitis A virus, hepatitis E virus, *Yersinia* sp., *Campylobacter* spp., *Salmonella* spp., *Trichinella* sp., *Toxoplasma* sp., *Cryptosporidium* sp., *Giardia* sp., *Taenia solium*, *Staphylococcus aureus*, *Clostridium botulinum*, *Clostridium perfringens*, *Escherichia coli*, *Shigella* sp., *Vibrio* sp., *Listeria monocytogenes*, and *Bacillus cereus* [5,13,17].

## 3. Fish and Products

Fish are any food extracted from aquatic environments intended for human or animal consumption; they are considered a generic term and include fish, mollusks, crustaceans, and algae, among other organisms [2]. In 2020, 177.8 million tons of fish supplied for human consumption were produced from capture fishing (90.3) and aquaculture (87.5) activities, with a per capita consumption of 20.2 kg, where live, fresh, or refrigerated products were the most commonly used for direct human consumption, followed by frozen, prepared, preserved, and cured products [18].

The consumption of fish and animal proteins has been increasing for several years, and it is estimated that this trend will continue around the world, especially in industrialized countries, mainly because of the wide variety and availability of products in commerce and where aquaculture has the capacity to satisfy the increasing demand for animal proteins [19].

Fish, as food, have a high proportion of high biological value and digestible proteins, as well as unsaturated lipids, vitamins, minerals, a high water content, and a pH close to neutral [2]. Therefore, in addition to factors such as the formulation of the product, the conservation process applied, and the sanitary conditions of processing, packaging, storage, distribution, and handling practices influence its stability, spoilage, shelf life, and health risk for consumers [20].

Human diseases caused by pathogenic microorganisms transmitted by fish and products can be derived from handling and/or consumption, with influencing factors such as the time of the year, the contact of patients with fish and related environments, eating habits, and the state of the individual’s immune system [3]. For fish and shellfish, some of the causal agents of diseases frequently related to consumption are *Diphyllobothrium latum*, *Clonorchis sinensis*, *Opisthorchis* spp., *Anisakis* sp., *Bacillus cereus*, *Listeria* spp., *E. coli*, *Salmonella* spp., *S. aureus*, *Vibrio* spp., *Aeromonas* spp., biogenic amines (histamines), and biotoxins [1,8,13,16,21].

## 4. Fish Microbiology

The microbiological profile of fish depends mainly on the microorganisms present in the aquatic environment where they live, in addition to other factors such as eating habits, age, species, season of the year, water temperature, handling conditions, capture methods in fishing, and medications used in aquaculture activities [3,22,23,24,25]. While living, freshly caught or farmed, microorganisms are located on external surfaces such as the skin and gills and in the intestines, resulting in a variation in the total number of microorganisms, with an established normal range of 10^2^ to 10^7^ CFU/cm^2^ on the surface of the skin, whereas for the gills and intestines, the number ranges from 10^3^ to 10^9^ CFU/g [22].

The edible portion (muscle) of healthy, freshly caught fish is typically sterile because of the activity of its immune system. Once a fish has died, its immune system collapses, and the bacteria present or contaminants can proliferate mainly on the surface and freely invade the muscle, penetrating between muscle fibers [22,26], which is associated with spoilage and health risk upon consumption [3,22,24,27]. The bacteriological profile of fish can vary and consists of gram-negative bacteria such as *Pseudomonas*, *Moraxella*, *Acinetobacter*, *Shewanella*, *Flavobacterium*, *Vibrio*, and *Photobacterium* (typical of marine fish), *Plesiomonas* sp., *Aeromonas* spp. (typical of freshwater fish), and gram-positive bacteria such as *Bacillus*, *Micrococcus*, *Clostridium*, *Lactobacillus*, and *Coryneformes*, which can be found in different proportions [22,25]. On the other hand, in fish from highly contaminated waters, high counts of 10^7^ CFU/cm^2^ can be found [22], and these bacteria are mainly members of the *Enterobacteriaceae* family [22,26].

In terms of quality, spoilage, and safety, the bacteria present in fish are classified as the following: (1.) native bacteria, such as *Clostridium botulinum*, *Vibrio* spp., *Plesiomonas* sp., and *Aeromonas* spp., that naturally inhabit aquatic environments where the water temperature has a selective effect and (2.) non-native bacteria, including various enterobacteria, such as *Proteus* sp., *Citrobacter* sp., *Serratia* sp., *Escherichia coli*, *Shigella* sp., *Salmonella* spp., *Klebsiella* spp., and gram-positive bacteria, such as *Staphylococcus aureus* and *Listeria monocytogenes* originating from aquatic environments contaminated with fecal matter or derived from handling, transportation, unhygienic storage, and/or the use of contaminated water in the processing and cleaning of fish [3,24,26,27].

## 5. *Klebsiella* spp.

Members of the genus *Klebsiella* spp., are microorganisms belonging to the *Enterobacteriaceae* family, which have a bacillus morphology; are not sporulated; are immobile; are facultative anaerobes, chemoorganotrophs, oxidase-negative, and gram-negative; have nitrate to nitrite reducers; ferment lactose and glucose mainly to 2,3-butanediol, ethanol, CO_2_ and H_2_; and do not produce H_2_S [28,29,30,31,32,33]. They are between 0.3 and 1.5 µm in diameter and 0.5 and 6.0 µm in length; they have an optimal growth temperature between 30 and 37 °C and are at pH 7.2. The percentage of guanine plus cytosine (GC) is 53–58 mol%, and they have no nutritional requirements for their growth [24,29,32,34,35]. These bacteria generally have a polysaccharide capsule that, when observed in solid culture, presents mucoid colonies of viscous consistency [24,29,32]. In the genus *Klebsiella*, there are the species *K. pneumoniae* ssp. *pneumoniae*, *K. pneumoniae* ssp. *rhinoscleromatis*, *K. pneumoniae* ssp. *ozaenae*, *K. oxytoca*, *K. planticola*, *K. terrigena*, and *K. ornithinolytica* [35], where *K. pneumonia*, *K. granulomatis* (formerly *Calymmatobacterium granulomatis* and proposed reclassification) [35,36], and *K. oxytoca* have been mostly associated with opportunistic infections in animals and humans [32]. *Klebsiella* infections have been reported in soft tissue and the urinary tract, as well as in septicemia, chronic atrophic rhinitis, enteritis, arthritis, diarrhea, and pneumonia, and they occur mainly in hospitals and cause severe morbidities and mortality [24,28,32,37,38]. These microorganisms present various virulence factors that contribute to their pathogenicity, such as the capsule with antigen “K”, which is the most important factor and protects bacteria from phagocytosis by polymorphonuclear cells; strains lacking a capsule are less pathogenic than those that have it; another factor is the cell surface lipopolysaccharide with somatic antigen “O”, the iron uptake and exchange systems, fimbrial adhesins, cytotoxins, and hemolysins [24,28,34].

*Klebsiella* sp., inhabit different environments, forming part of the microbiota of soil, plants, fresh and saltwater bodies, sediments, animals, and humans by colonizing the skin, mucous membranes, nasal epithelium, gastrointestinal tract, and feces [24,28,32,33,37,39,40,41,42].

In humans, the gastrointestinal tract is a reservoir for *Klebsiella*, making it an important vehicle of transmission through food handlers in community or hospital environments [29,38]. Moreover, in bodies of water and fish, since *Klebsiella* spp., is a member of the microbiota, it is considered a zoonotic agent, along with enterobacteria such as *Escherichia coli* and *Salmonella*, where an infection can occur through direct contact by wounds and consumption of contaminated water and/or food [24,41].

### 5.1. Klebsiella pneumoniae

Within the genus, *Klebsiella pneumoniae* is one of the main causes of a variety of infections in humans and animals in both community and hospital environments, including conditions such as pneumonia, urinary tract infections, wound infections, liver abscesses, bacteremia, diarrhea, and extraintestinal pathological processes [6,33,40,43,44], and affects immunocompromised patients, elderly individuals, newborns, and intensive care unit patients in hospitals [29,44].

On the other hand, the isolation of this bacterium has been reported in various foods, such as raw meat, raw vegetables, fruit juices, fish, crustaceans, powdered infant formula, street foods, and ready-to-eat foods, making it an important foodborne pathogen [40,41,43] that is frequently linked to healthy people as carriers, and food- and meat-producing animals as transmitters [6,40].

*K. pneumoniae* presents several virulence factors associated with the development of the disease, such as capsules, endotoxins, siderophores, iron elimination systems, and adhesins. The capsule is relevant to protection against phagocytosis and inhibition of the host immune response [43]. Some capsular types (K), such as K1, K2, K5, K20, K54, and K57, are often associated with community-acquired invasive pyogenic liver abscess syndrome, septicemia, and pneumonia, whereas K1, K2, K20, K54, and K57 are frequently related to infections in humans and animals [43,44]. Notably, for several years, the emergence of a hypervirulent variant of *Klebsiella pneumoniae* (hvKp) with respect to classical *K. pneumoniae* (cKp) has been reported [45,46]. This variant, as its name indicates, is more virulent, causing community-acquired infections, frequently in healthy individuals with high morbidity and mortality rates [45,47].

Owing to its virulence, hvKp has several virulence determinants, including siderophore systems for iron acquisition, increased capsule production, K1 and K2 capsule types, and the colibactin toxin. Additionally, hvKp strains phenotypically display hypermucoviscosity, a distinctive feature of isolates under laboratory conditions due to increased production of capsular polysaccharides [46,47].

hvKp can be carried in the mouth, skin, and gastrointestinal tract, which contributes to its spread in community and hospital settings, generating a variety of conditions, such as liver abscesses, with the ability to metastasize from one organ to another, even in distant sites, such as the eyes, lungs, and central nervous system. This characteristic of the variant distinguishes it from cKp; it is also related to primary extrahepatic infections, bacteremia, pneumonia, and soft tissue infections [46,47], and the emergence of multidrug resistance has been reported, which is why interest and concern in public health worldwide has been increasing [45,46,47].

### 5.2. Association of Klebsiella with Animal Health: Aquaculture Production

Like all animals, fish are susceptible to diseases both in their natural environment, such as rivers, streams, lakes, and reservoirs, as well as in their production through aquaculture activities, with the latter having a greater impact due to population densities during production [48].

In aquaculture farms, unfavorable environmental conditions such as poor physical–chemical water quality (temperature, pH, high ammonium levels, and low oxygen levels), poor production practices such as incorrect handling, inadequate nutrition, overcrowding, underfeeding or overfeeding can stress fish, causing a reduction in growth rate; inhibition of the immune system; and susceptibility to diseases caused by bacteria, viruses, fungi, or parasites, which can cause mortality and economic losses [39,48].

In terms of environment and aquaculture activities, bacteria of the genera *Klebsiella*, *Proteus*, *Serratia*, *Enterococcus*, *Pseudomonas*, *Plesiomonas*, *Enterobacter*, *Staphylococcus*, *Acinetobacter*, *Stenotrophomonas*, *Kocuria*, *Micrococcus*, *Kluyvera*, *Pandoraea*, and *Edwarsiella* are present as part of the microbiota of fish or aquatic ecosystems, but they can become opportunistic pathogens when there are stressful factors affecting the fish, with tissue infections, hemorrhages in internal organs and reduced immunity in fish prevailing [3,33,39,49].

Species of the genus *Klebsiella* can infect a variety of fish. Adeshina [32] reported that, in aquaculture activities, *Klebsiella* spp., has been isolated from the gills, skin, intestine, liver, and muscle of catfish, accounting for 33.33 to 36.59%, and is related to infection of the animals in culture, possibly due to an imbalance in the environmental and physiological conditions of the fish. As a result, the fish and products are considered threats to the health of consumers.

*K. pneumoniae* reportedly infects and causes disease outbreaks in a variety of aquatic animals, such as *Labeo rohita*, *Amphiprion nigripes*, *Hirudo nipponia*, *Nemipterus japonicus*, *Nishikigoi carp*, and *Cyprinus carpio* [33,50]. It causes hemorrhages, red spots along the body, skin discoloration with ulceration in *Nishikigoi carp* and *Cyprinus carpio*, exophthalmia in *Nemipterus japonicus* [33], and hemorrhages, ulcers, skin redness, and mortality in *Amphiprion nigripes* [33,39]. In Nile tilapia, it causes lethargy, anorexia, subcutaneous hemorrhages, urogenital hemorrhage, ascites, hepatomegaly, splenomegaly, gill lesions, cloudy eyes, bilateral exophthalmia, ocular hemorrhage, and mortality [33,45].

Animal health in aquaculture and fishery production is related to food safety since its detrimental effects favor the risk of zoonoses, which have received increasing attention in terms of public health due to factors such as their incidence or the identification of new zoonotic agents in animals intended for human consumption, such as fish [45].

### 5.3. Klebsiella in Fish and Antimicrobial Resistance

In terms of human health, antibiotics, such as ampicillin, sulfonamides, tetracycline, trimethoprim–sulfamethoxazole, ciprofloxacin, nalidixic acid, nitrofurantoin, tobramycin, gentamicin, amikacin, ticarcillin/clavulate, imipenem, aztreonam, and third- and fourth-generation cephalosporins, have been reported to be useful for the treatment of *Klebsiella* infections. Drug susceptibility testing is essential for drug administration [28,51]. However, the treatment of infections through the application of antibiotics is limited because the causative agent is resistant to multiple antibiotics, making it a greater challenge to recover health, especially in immunocompromised patients [24].

Antimicrobial resistance caused by microorganisms is considered a global public health problem worldwide [52,53] because of economic impacts such as the cost of longer hospital stays, the need to search for effective and expensive medications, financial difficulties for affected people, and the generation of more cases of death and disability [53].

In 2019, approximately 5 million human deaths worldwide were linked to antimicrobial resistance, including approximately 1.27 million directly due to resistant bacteria, the main pathogens of which are *Klebsiella pneumoniae*, *Escherichia coli*, *Staphylococcus aureus*, *Streptococcus pneumoniae*, *Acinetobacter baumannii*, and *Pseudomonas aeruginosa* [52]. The widespread, improper, and excessive use of antibiotics in agricultural, livestock, and aquaculture activities, as well as for the treatment of human infectious diseases, has led to the emergence and spread of a variety of multidrug-resistant bacteria, including pathogenic bacteria, in the environment [3,54,55].

In food production, contamination by antibiotic-resistant microorganisms represents a global threat to public health through impacts on food safety [55,56]. Food has been identified as an important medium for the development of and vehicle for the transmission of microbes resistant to various antibiotics to humans; additionally, direct contact with affected animals or the consumption of contaminated food has led to the acquisition of infections and diseases that are difficult to treat [40,55,56]. Therefore, the presence of antimicrobial-resistant microorganisms in the food chain is considered a potential route of exposure for the entire population [56].

Resistance by bacteria can be a natural property (intrinsic) or acquired by mutation or exchange of genetic material (plasmids or transposons) through various mechanisms, such as transduction, conjugation, transformation, and transposition [57]. Several mechanisms of acquired resistance to antimicrobials have been reported: (1.) enzymatic modification or destruction of the antibiotic; (2.) changes in membrane permeability; (3.) alteration or production of new target sites; (4.) the presence of efflux pumps that expel the antibiotic [57,58], where bacteria can use more than one mechanism [57], and gram-negative bacteria, such as *Klebsiella*, tend to predominantly use enzymatic modification of the antibiotic with the generation of β-lactamases. The generation of extended-spectrum β-lactamases (ESBLs), which confer resistance to third-generation cephalosporins and carbapenems that are mediated by plasmids, has been reported, resulting in a high capacity for dissemination among gram-negative species, which is frequently reported in *Klebsiella pneumoniae* and *E. coli* [40,58]. As a result, species such as *Klebsiella pneumoniae* have been classified by the World Health Organization as antimicrobial-resistant microorganisms of great interest in health and priority pathogens on the list of antibiotic-resistant pathogens that require new treatments [40].

In food production by aquaculture and fishing activities, aquatic environments are a considerable factor in the dissemination of antimicrobial resistance by receiving chemical or microbiological contaminants from industrial, domestic, agricultural, livestock, and/or aquaculture activities. They can act as promoters of genetic exchange and contribute to the dissemination of resistance between microorganisms present in the environment and aquatic animals, many of which are zoonotic in nature [54,59]. On the other hand, in aquaculture activities, fish subjected to overcrowding and stress conditions are more susceptible to microbial infections, which increases the use of antimicrobial agents prophylactically and/or therapeutically, contributing to the development and dissemination of resistance [60].

Several studies around the world have reported the isolation of various enterobacteria, including species of the genus *Klebsiella* spp., which are resistant to various antimicrobials in marketed fish intended for human consumption. Thongkao and Sudjaroen [59] reported the isolation of penicillin- and ampicillin-resistant *K. pneumoniae* strains from tilapia (*O. niloticus*) from aquaculture and marketing activities in Asian countries such as Thailand. Notably, antimicrobial resistance is attributed to the repeated and indiscriminate use of antibiotics in these activities. Marijani [3] reported that *Klebsiella* spp., was isolated from marine and freshwater fish marketed in Tanzanian markets, with a prevalence of up to 28% and resistance to six antimicrobials (gentamicin, tetracycline, penicillin, erythromycin, azithromycin, and ciprofloxacin). Matyar [54] noted the presence of *Klebsiella oxytoca*, *Klebsiella pneumoniae* ssp. *pneumoniae*, and *Kluyvera cryocrescens* in fish gills (*Sea bream*, *Sparus aurata*; *Red mullet*, *Mullus barbatus*; *Smelt*, *Atherina boyeri*, *Sardina pilchardu*, *Sand steenbras*, *Lithognathus mormyrus*) from the Mediterranean and Aegean Seas showing resistance to several antibiotics, mainly penicillin (ampicillin), cephalosporins (cefazolin), and tetracyclines.

Cerdeira et al. [61] identified multidrug-resistant (ceftriaxone, cephalothin, cefotaxime, aztreonam, tetracycline, sulfonamide, gentamicin, ciprofloxacin, and nalidixic acid) extended-spectrum β-lactamase (ESBL)-producing *Klebsiella pneumoniae* strains isolated from native freshwater catfish marketed for human consumption on the market in the Amazon region of Brazil. They concluded that there is a risk to human health and that contamination of aquatic environments by aquaculture activities and domestic, agricultural, industrial, and hospital discharges have contributed to the spread of bacteria and resistance genes around the world. Furthermore, the aquaculture or fishery product market itself can be a source of resistant pathogens that could result from poor handling, storage, and transport practices that can predispose fish to contamination, posing a hazard to food safety.

Rehman et al. [62] reported a microbiological analysis of three species of raw fish (*Labeo rohita*, *Catla catla*, and *Cirrhinus mrigala*) marketed in Pakistan from retail markets and ponds. The prevalences of *Klebsiella pneumoniae* were 15 and 13.3%, and the prevalences of *Klebsiella oxytoca* were 10 and 8.3% in market and pond fish samples, respectively. These microorganisms are resistant to norfloxacin, cefoxitin, bacitracin, and erythromycin, indicating a risk to the health of the population and that the continuous use of antibiotics in aquaculture leads to the development of microorganisms that are resistant to antimicrobials, forming reservoirs for resistance genes, and the transfer of resistant organisms from the aquaculture environment to the natural aquatic environment may occur. Krahulcova et al. [60] determined the presence of various bacteria, including *Klebsiella* spp., which are resistant to gentamicin, ampicillin, and chloramphenicol, in fish, seafood, and products from markets and restaurants in Slovakia. They concluded that fish and seafood distributed and marketed for consumption present resistant microorganisms with a risk of generating foodborne diseases throughout the food chain, which leads to the implementation of better management and control of food sanitation.

Isolation in aquaculture farms, associated with fish pathologies, zoonotic nature, presence in food, resistance to multiple antibiotics (becoming a potential reservoir of antibiotic resistance genes), and foodborne transmission of some members of the genus, such as *K. pneumoniae*, have been reported and have begun to raise concerns in terms of human health [40,41].

### 5.4. Klebsiella and Biogenic Amines in Fish for Human Consumption

Microbial activity is strongly associated with fish spoilage, particularly protein breakdown in muscle, which results in the generation of various metabolites, such as biogenic amines (histamines, putrescine, cadaverine, tyramine, agmatine, and tryptamine), from amino acids, such as histidine, ornithine, lysine, tyrosine, arginine, and tryptophan, which are derived from the activity of decarboxylating enzymes, frequently being histamine, and are the cause of food poisoning, mainly after the ingestion of fish from the *Scombridae* family (tuna, bonito, mackerel) [5,8,63,64]. The enzyme histidine decarboxylase produces histamine from histidine; this enzyme is produced by several *Enterobacteriaceae*, such as *Klebsiella pneumoniae*, *Klebsiella oxytoca*, *Vibrio alginolyticus*, *Aeromonas hydrophila*, *Enterobacter cloacae*, *Enterobacter aerogenes*, *Clostridium perfringens*, *Bacillus* spp., *Pseudomonas* spp., *Proteus* spp., *Serratia marcescens*, *Morganella morganii*, and *Hafnia alvei* [1,8,39,63,64,65]. Histamine-producing microorganisms proliferate in contaminated food when it is stored in the absence of refrigeration or under inadequate refrigeration conditions. Therefore, hygiene and temperature conditions from capture, processing, and storage are important factors in controlling and preventing histamine-producing enzyme activity, which is favored when fish are stored at temperatures above 4.4 °C [8,64]. The food safety regulations of the European Union and the United Mexican States establish that the maximum permissible histamine content for fish and products (species associated with a high histidine content) is 100 mg/kg, whereas that for the United States Food and Drug Administration (USFDA) is 50 mg/kg [63].

## 6. Microbiological Analysis of Fish and Products

Since *Klebsiella* spp., belongs to the *Enterobacteriaceae* family, it has simple nutritional requirements for its isolation and identification from food, which is carried out using nutritive or enrichment culture media and differential and selective media commonly used in the laboratory. Owing to its ability to ferment lactose, the color of the colonies during growth varies according to the culture medium used. Salmonella–Shigella agar (red or pink colonies), McConkey agar (mucoid red or pink colonies), methylene blue eosin agar (large mucoid, brown or gray colonies with black centers), cystine lactose electrolyte-deficient agar (distinctly yellow colonies), bismuth sulfite agar, Drigalski agar, nutrient broth, tryptone soy broth, blood agar, chocolate agar, Mossel EE broth, brilliant green bile lactose, chromogenic media containing substrates for the detection of ß-glucosidase, and various morphological and biochemical identification tests (Table 1) can be used in relation to the rest of the enteric bacteria, including the Gram stain, catalase tests, motility tests, oxidase tests, indole tests, glucose fermentation products (the methyl red test and Vogas–Proskauer test), lactose fermentation, malonate utilization, citrate utilization, and urease tests [28,30,31,33,34,35,40,66,67].

On the other hand, *Klebsiella* sp., is considered part of the microbial group of coliforms along with the genera *Escherichia* sp., *Citrobacter* sp., and *Enterobacter* sp., as they share biochemical features such as being gram-negative, facultative anaerobes, lactose fermenters to lactic acid, gas generators, a regular habitat in the human and animal intestine, soil, sewage, and organic waste. This indicator microbial group is related to the hygienic qualities of water and food and the pre- and post-production conditions to which they are subjected. Its presence in water and food represents a risk of disease when consumed [66,67].

**Figure 1:**
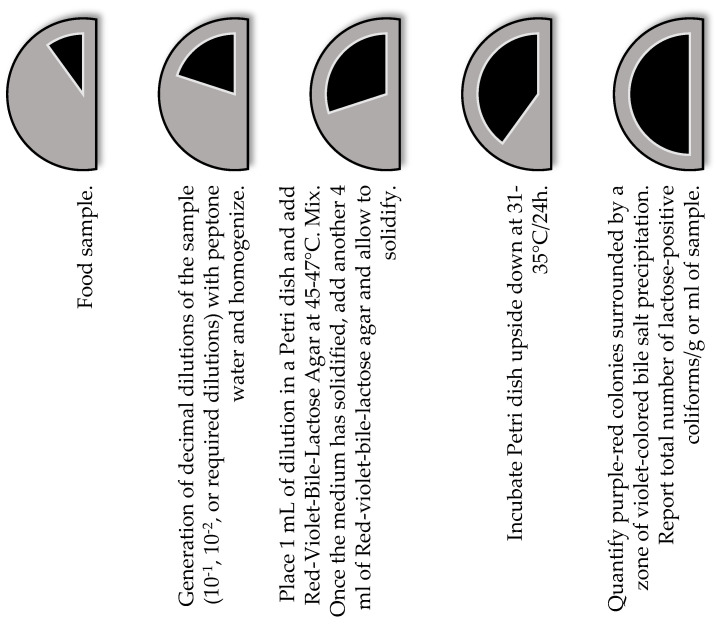
Methods for the identification and quantification of total positive coliforms for lactose in foods [66,68].

**Figure 2:**
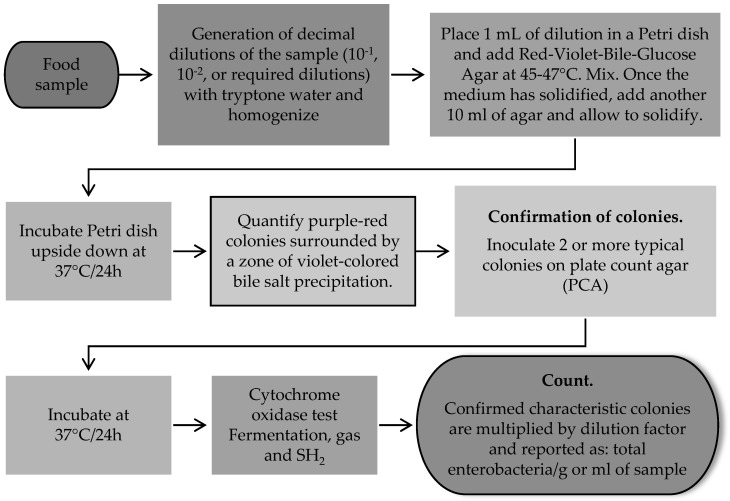
Methods for the isolation and determination of total enterobacteria in foods [66].

**Figure 3:**
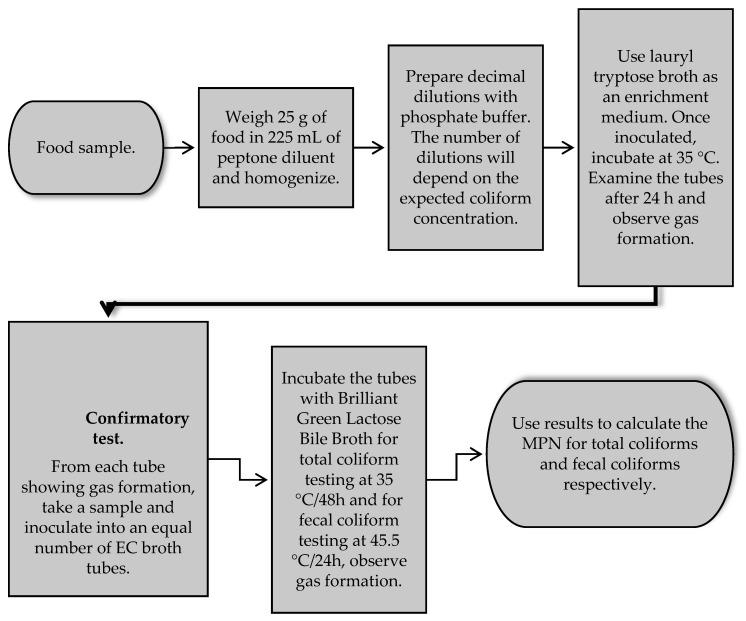
Determination of total and fecal coliforms according to the most probable number (MPN) [72].

Different methods have been developed and proposed around the world for the detection and enumeration of enterobacteria in foods to detect and isolate members of the genus *Klebsiella* spp. These methods use selective and differential culture media for the detection and quantification of total lactose-positive coliforms [66,68] (Figure 1) and total enterobacteria [66] (Figure 2). Food safety regulatory agencies, such as the Food and Drug Administration of the United States of America (US FDA), use differential and selective media for the determination and isolation of coliforms and *E. coli* in foods through the most probable number (MPN) method and growth in a solid medium using violet red bile agar [69]. In addition, the method proposed by the International Standards Organization (ISO) ISO 21528-1: 2017 [70] for the detection and enumeration of enterobacteria uses the most probable number and ISO 21528-2: 2017 [71] in the isolation and counting of enterobacteria colonies on plates. In Latin American countries such as Mexico, for the analysis of food safety, the official standard NOM-113-SSA1-1994 [68] establishes a method for counting total coliform microorganisms in a Petri dish from food products, indicating that the presence of these microorganisms may involve poor sanitary practices in the handling and manufacturing of food, low efficiency of sanitary and hygienic practices of the equipment, low microbiological quality of water and ice used in food processing areas and the risk of illness due to the presence of pathogens (Figure 1). Moreover, the official standard NOM-210-SSA1-2014 [72] establishes a method for estimating the density of total coliforms, fecal coliforms, and *E. coli* present in food for human consumption and water by the MPN method. The aforementioned methods involve the use of selective growth and enrichment culture media such as lauryl tryptose broth, brilliant green bile lactose broth, and *Escherichia coli* (EC) broth. The MPN method consists of presumptive and confirmatory testing using a series of three, five, and ten tubes depending on the expected contamination and the degree of accuracy desired. The principle of this method is based on the dilution of the sample in a series of multiple tubes, where the tubes of the lowest dilution are positive, and all the tubes of the highest dilution are negative. The positive result will be the presence of gas and growth of total coliforms by fermentation of lactose at 35 °C and fecal coliforms at 45.5 °C in foods (Figure 3) [72].

**Figure 4:**
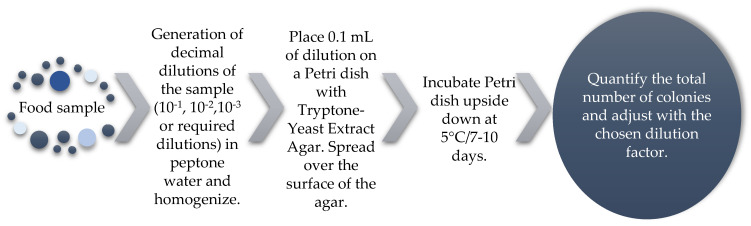
Analysis of psychrophiles in foods [66].

Similarly, *Klebsiella* belongs to a group of psychrotrophic organisms, along with other genera, such as *Flavobacterium*, *Bacillus*, *Pseudomonas*, *Serratia*, *Aeromonas*, *Proteus*, *Staphylococcus*, *Listeria*, *Yersinia*, and *Enterobacter*, as they are capable of growing at low temperatures. This is why this group of microorganisms is of interest in the storage, spoilage, and safety of food under refrigeration conditions (0–6 °C) [66,73], and methods have been developed for its isolation and quantification (Figure 4).

**Figure 5:**
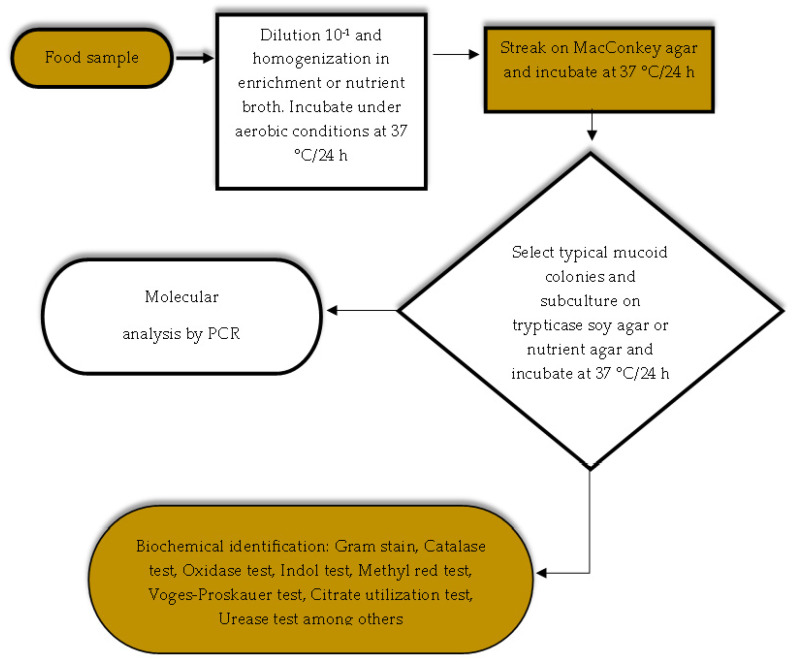
Method for the isolation and detection of *Klebsiella pneumoniae* in food [40,43].

On the other hand, owing to the great similarity between some genera and species and the complexity and microbial diversity of samples, the identification of *Enterobacteria* is usually a challenge despite traditional microbiological analyses [34,74]. Therefore, complementary or alternative methods based on the genome may be carried out in a more precise and shorter time than traditional methods [34]. Specific methods have been reported for the isolation and detection of species of sanitary interest, such as *K. pneumoniae* in food using enrichment, selective and differential culture media, subculture on nutrient agar for biochemical identification and confirmation via polymerase chain reaction (PCR) [40,43] (Figure 5). Detection by molecular methods is based on polymerase chain reaction of the different variants as target genes associated with virulence factors, such as *fimH*, *ureA*, *uge*, *wabG*, *magA*, and *Kfu*; the specific *16S rRNA* gene; the *GyrA* gene [31,33,37,39,43,45]; and the *gapA* gene, which are responsible for synthesizing the glyceraldehyde 3-phosphate dehydrogenase protein [24].

Another rapid and effective alternative to the identification of enterobacterial isolates, such as *Klebsiella* sp., originating from or associated with fish, seafood, and products by traditional or genomic means, is matrix-assisted laser desorption/ionization time-of-flight mass spectrometry (MALDI-TOF MS), which is based on the fact that each bacterium has a particular fingerprint of protein expression [61,74,75,76].

## 7. Prevention of Diseases Caused by Fish Production and Consumption

The production and exchange of food and services between countries around the world have led to the spread and harmonization of knowledge and basic actions throughout the food chain that allow the acquisition, availability, and consumption of food without contamination, which may lead to various infections or poisonings [1,77].

Fish are generally consumed after cooking and can be a low-risk food for health. However, the trend of consuming raw or ready-to-eat fish is increasingly common; therefore, this increases the risk of contamination in the processing, preparation, and service stages of food by various disease-causing agents, which can also present resistance to antimicrobials and have serious ecological and public health implications [54].

At the global level, regulatory frameworks for the control or prevention of pathogenic microorganisms in foods that cause diseases guarantee food safety, but only in foods and microorganisms with established limits [6]. However, there are various microorganisms whose health interest is increasingly relevant, such as *Klebsiella* spp. Several countries around the world, such as Mexico, with fishing, aquaculture, and processing activities, do not have a regulatory framework for food safety specific to these emerging pathogens in food. The identification of new hazards through food, where exposure or the appearance of new or increased susceptibilities to a known hazard is generated, is important to prevent or control emerging hazards, promote research, improve knowledge in the scientific community, and subsequently, the dissemination of information to all members of the food chain, including consumers [6].

The contamination, spoilage, and health risks of fish and products by various microorganisms, including *Klebsiella* spp., are generally related to the hygiene conditions and practices applied in the aquaculture or fishing environment, handling, conservation (temperatures above 4 °C favor microbial growth and the formation of histamines), and insufficient cooking or postcooking contamination [32,40,59,78,79].

The safety of fish and their products throughout the food chain is promoted through the application of various tools that are also useful for combating antimicrobial resistance, such as good practices in fishing and aquaculture (the loss of animal health in most cases is associated with practices that generate stress in the organisms, whether nutritional, environmental, or due to densities of culture organisms), good manufacturing practices, standardized operating procedures for sanitation (SSOPs) and the implementation of hazard analysis and critical control points (HACCPs), and food hygiene education for aquaculturists, fishermen, marketers, handlers, and end consumers (hand cleaning, separation of raw and cooked foods, complete cooking, food preservation at safe temperatures, use of safe water and raw materials, among others [1,32,48,56,59,77,80,81,82]).

## 8. Conclusions

Fish are highly produced, marketed worldwide, very nutritious and considered essential components of the human diet. However, fish are also products that are very susceptible to deterioration and contamination by microorganisms throughout the food chain, as they are foods with a short shelf life and a risk of foodborne illnesses whose causal agents are usually bacteria.

*Klebsiella* spp., are microorganisms of global importance in human and animal health, as they are opportunistic pathogens associated with diseases and mortality in aquaculture and various human infections and poisonings due to fish consumption. These bacteria are considered cosmopolitan and are constituents of the natural microbiota of fish or external contaminants derived from inadequate hygiene conditions and practices in the primary production and postcapture stages (handling, processing, and marketing, among others). This leads to this food being considered a source of transmission and risk to human health.

The approach and promotion of the exploration and knowledge of the *Klebsiella* genus and its association with food are becoming increasingly interesting since these microorganisms are frequently related to community and nosocomial infections, present multiresistance to antibiotics, and make treatment difficult. Considering that the world population, at-risk populations, food demand, new production technologies, and product supply are increasing, sanitary interest in fish, products, and their biological hazards is increasingly noticeable. Thus, research on this pathogen and related foods has led to greater development, understanding, and dissemination, all in favor of the protection of public health.

## Figures and Tables

**Table 1:** Analysis tests for the biochemical identification of the *Klebsiella* genus in relation to other enterobacteria and members of the coliform indicator group [30].

Bacterial Genus		Test									
	Indole	Mobility	Gas *	H_2_S	Urease	VP	β-gal	Citrate	MR	Alanine Deaminase	KCN
***Klebsiella***	−	−	+	−	+	+	+	+	−	−	+
***Escherichia***	+	+ or −	+	−	−	−	+	−	+	−	−
***Enterobacter***	−	+	+	−	−	+	+	+	−	−	+
***Yersinia***	−	+	−	−	+	−	+	−	+	−	−
***Citrobacter***	−	+	+	+ or −	−	−	+	+	+	−	+ or −
***Providencia***	+	+	−	−	−	−	+	+	+	+	+

* From glucose. β-gal: β-galactosidase. VP: Voges–Proskauer. MR: methyl red. KCN: potassium cyanide. +: positive −: negative.

## Data Availability

All data generated or analyzed during this study are included in this published article.
